# The Pine River Statement: Human Health Consequences of DDT Use

**DOI:** 10.1289/ehp.11748

**Published:** 2009-05-04

**Authors:** Brenda Eskenazi, Jonathan Chevrier, Lisa Goldman Rosas, Henry A. Anderson, Maria S. Bornman, Henk Bouwman, Aimin Chen, Barbara A. Cohn, Christiaan de Jager, Diane S. Henshel, Felicia Leipzig, John S. Leipzig, Edward C. Lorenz, Suzanne M. Snedeker, Darwin Stapleton

**Affiliations:** 1 School of Public Health, University of California, Berkeley, California, USA; 2 Wisconsin Division of Public Health, Madison, Wisconsin, USA; 3 Department of Urology, University of Pretoria, Pretoria, South Africa; 4 School of Environmental Sciences and Development, North-West University, Potchefstroom Campus, Potchefstroom, South Africa; 5 School of Medicine, Creighton University, Omaha, Nebraska, USA; 6 Public Health Institute, Oakland, California, USA; 7 School of Health Systems and Public Health, University of Pretoria, Pretoria, South Africa; 8 School of Public and Environmental Affairs, Indiana University, Bloomington, Indiana, USA; 9 Pine River Superfund Citizen Task Force, St. Louis, Michigan, USA; 10 Center for Responsible Leadership and; 11 Public Affairs Institute, Alma College, Alma, Michigan, USA; 12 Sprecher Institute for Comparative Cancer Research, Cornell University, Ithaca, New York, USA; 13 Emeritus, Rockefeller University, New York, New York, USA

**Keywords:** DDE, DDT, dichlorodiphenyldichloroethylene, dichlorodiphenyltrichloroethane, health effects, organochlorine pesticides, persistent organic pollutants

## Abstract

**Objectives:**

Dichlorodiphenyltrichloroethane (DDT) was used worldwide until the 1970s, when concerns about its toxic effects, its environmental persistence, and its concentration in the food supply led to use restrictions and prohibitions. In 2001, more than 100 countries signed the Stockholm Convention on Persistent Organic Pollutants (POPs), committing to eliminate the use of 12 POPs of greatest concern. However, DDT use was allowed for disease vector control. In 2006, the World Health Organization and the U.S. Agency for International Development endorsed indoor DDT spraying to control malaria. To better inform current policy, we reviewed epidemiologic studies published from 2003 to 2008 that investigated the human health consequences of DDT and/or DDE (dichlorodiphenyldichloroethylene) exposure.

**Data sources and extraction:**

We conducted a PubMed search in October 2008 and retrieved 494 studies.

**Data synthesis:**

Use restrictions have been successful in lowering human exposure to DDT, but blood concentrations of DDT and DDE are high in countries where DDT is currently being used or was more recently restricted. The recent literature shows a growing body of evidence that exposure to DDT and its breakdown product DDE may be associated with adverse health outcomes such as breast cancer, diabetes, decreased semen quality, spontaneous abortion, and impaired neurodevelopment in children.

**Conclusions:**

Although we provide evidence to suggest that DDT and DDE may pose a risk to human health, we also highlight the lack of knowledge about human exposure and health effects in communities where DDT is currently being sprayed for malaria control. We recommend research to address this gap and to develop safe and effective alternatives to DDT.

Dichlorodiphenyltrichloroethane (DDT) is a potent insecticide that was used worldwide for agricultural and public health purposes from the 1940s until the 1970s, when concern about its toxic effects on wildlife and humans, its environmental persistence, and its concentration in the food supply led to restrictions and prohibitions on its use [Agency for Toxic Substances and Disease Registry (ATSDR) 2002]. Commercial mixtures, often called technical-grade DDT, contain two major isomers, the active ingredient, *p*,*p*′-DDT, and a by-product, *o*,*p*′-DDT. DDT and its primary breakdown product, dichlorodiphenyldichloro-ethylene (DDE), are highly lipophilic, persist in the environment, and bioaccumulate in humans because of their long half-lives (6 years and possibly up to 10 years, respectively) (Longnecker 2005; Wolff et al. 2000).

DDT was identified as a potent insecticide in 1939 and was heavily used during World War II. After the war, DDT became the global insecticide of choice in households, for agriculture, and for public health vector-control projects. In 1962, Rachel Carson, in *Silent Spring*, noted that DDT bioaccumulates and biomagnifies up the food chain and raised concerns that the pesticide may have long-lasting effects on wildlife and possibly on humans (Carson 1962).

In the United States, all nonpublic health uses of DDT were banned by 1972. Regulation by some other nations occurred more gradually. DDT continues to be used for malaria control in several African and Asian countries (Stockholm Convention on Persistent Organic Pollutants 2008). In 2001, more than 100 countries signed the Stockholm Convention on Persistent Organic Pollutants (POPs), committing to eliminate the use of 12 POPs of greatest concern to the health of the global community, including DDT (United Nations Environment Programme 2001). By 2008, 160 countries had ratified the Stockholm Convention, making it one of the most successful inter national environmental agreements (Stockholm Convention on Persistent Organic Pollutants 2008). Recognizing the continued need for DDT use in some countries, the convention allows the production and use of DDT for disease vector control only, provided that no safe, effective, and affordable alternatives are locally available. In these cases, the convention requires parties to notify the convention secretariat of their intention to produce and/ or use DDT for vector control and to prevent or minimize human exposure and release into the environment. In 2006, the World Health Organization (WHO) and the U.S. Agency for International Development (USAID) endorsed indoor DDT spraying to control malaria (WHO 2006).

On 14 March 2008, researchers met for the Eugene Kenaga International DDT Conference, which was jointly organized by the Pine River Superfund Citizen Task Force, the Center for Responsible Leadership, and the Public Affairs Institute of Alma College with the endorsement of the International Society of Environmental Epidemiology, the Society for Environmental Toxicology and Chemistry, Alma College, and the Pine River Superfund Task Force. The goal of the conference was to bring together experts on DDT and concerned citizens to address the current and legacy implications of DDT production and use. This conference was held at Alma College, near the Velsicol Chemical Corporation U.S. Superfund site in Gratiot County, Michigan.

The purpose of this review is to summarize information on health risks so as to better inform risk–benefit analyses and policy. We do so by reviewing evidence of human exposure to DDT and some of its potential health consequences, focusing primarily on studies that have been published since the endorsement of DDT by WHO and USAID and since the publication of other literature reviews on the subject (Longnecker 2005; Rogan and Chen 2005). We conducted a search of PubMed (National Library of Medicine, Bethesda, MD, USA) to find human studies (excluding case reports) published in English from 2003 to 2008 by using the following search terms: (DDT OR DDE) AND (toxicity OR health OR cancer OR carcinogenicity OR reproduction OR estrogen OR neurological OR development OR exposure OR diabetes OR pregnancy OR miscarriage OR spontaneous abortion OR birth weight OR gestation OR lactation OR birth defects OR growth OR puberty OR fertility OR neurotoxicity OR neurodevelopment OR immunological). We identified 494 papers and reviewed them for primary research. On the basis of available data, we make recommendations regarding the introduction, continuation, or reintroduction of DDT use worldwide. These recommendations represent the consensus opinion of the authors and participants present at the Eugene Kenaga International DDT Conference.

## Potential for Human Exposure

Environmental and biological monitoring studies in the United States demonstrate that use restrictions were successful in lowering human exposure to DDT. Estimated dietary intake of DDT dropped > 200% between 1970 and 1986 (ATSDR 2002), whereas serum DDT concentrations declined 9-fold between 1980 and 2000 [Centers for Disease Control and Prevention (CDC) 2003; Murphy et al. 1983]. Recent studies in the United States report low concentrations of DDT and DDE in food (U.S. Food and Drug Administration 2002) and in house dust and soil (Bradman et al. 1997; Butte and Heinzow 2002). Yet nearly all U.S. residents have measurable serum *p*,*p*′-DDE levels, whereas *p*,*p*′-DDT is detected in 5–10% of the population (CDC 2005).

A sample of recent studies of DDT/DDE serum levels in pregnant women and women of reproductive age shows the different exposure scenarios around the world. In this sample of studies, low concentrations were observed in U.S. women of reproductive age participating in the 2001–2002 wave of the National Health and Nutrition Examination Survey (NHANES; Table 1). The median DDE serum concentration was 10-fold higher in a population of primarily Mexican immigrant women living in an agricultural area of California where half of the women had immigrated within the previous 5 years (Bradman et al. 2007). DDE concentrations in this Mexican-American cohort were similar to those of a concurrent population living in Morelos, Mexico, where DDT probably had not been used for some time (Torres-Sanchez et al. 2007). However, median DDE serum concentrations in women residing in Chiapas, Mexico, where DDT may have been used up until 2000, were about five times higher in 1998 (Koepke et al. 2004) than in Morelos (Torres-Sanchez et al. 2007) and within the range observed in older studies from the United States when DDT was still being used (Bhatia et al. 2005; Gladen et al. 2004; Longnecker et al. 2005). However, the concentrations in pregnant women in Chiapas are still orders of magnitude lower than those reported in a study of South African men whose houses were sprayed with DDT as part of indoor residual spraying programs (mean blood DDE concentration = 239 ± 215 μg/g lipid) (Aneck-Hahn et al. 2007). Other studies conducted in South Africa also reported very high levels of DDT/DDE in breast milk (Bouwman et al. 1994, 2006). It may be that the pattern of use and/or formulation of DDT for malaria control differed in Chiapas and South Africa. Nevertheless, these data suggest that indoor residual spraying results in high DDT exposure in humans, including vulnerable populations, such as pregnant women and fetuses.

## Evidence for Carcinogenicity and Cancer in Humans

In 1991, the International Agency for Research on Cancer (IARC) rated DDT as “possibly carcinogenic to humans (Group 2B)” (IARC 1991). This rating was largely based on the induction of liver tumors in experimental animal studies that reported significant increases in hepatomas (neoplastic liver cell tumors) in multiple strains of male and female rodents exposed to technical DDT orally (gav-age or diet) or to *p*,*p*′-DDE (diet) (IARC 1991). Most human studies reviewed by IARC in 1991 did not show an association between DDT exposure and cancer risk. Some studies suggested that DDT exposure may be associated with certain cancers (lung cancer and lymphomas); however, the lack of control for exposure to other chemicals, small study size, insufficient data on confounding factors (e.g., incomplete information on tobacco use), and short follow-up time for long-latency cancers limited the ability to make any conclusions at that time (IARC 1991). Research on DDT/DDE exposure and cancer continued to yield mixed results after the publication of the IARC report. We review the recent research with a focus on cancers of the liver, pancreas, and breast.

### Liver cancer

In an ecologic study, Cocco et al. (2000) found that standardized mortality rates (SMRs) for liver cancer were elevated in whites but not in African Americans who lived in states with high population-level adipose tissue DDE concentrations. These researchers offered no explanation for this racial difference. In a case–control study, McGlynn et al. (2006) reported that the risk of liver cancer was significantly elevated in Chinese men with the highest blood levels of DDT [odds ratio (OR) = 3.8; 95% confidence interval (CI), 1.7–8.0] compared with men with lower levels of DDT. Blood levels of DDE were not associated with a higher risk.

### Pancreatic cancer

Mechanistic data suggest that DDT could play a role in pancreatic cancer by modulating activation of the oncogene *K-ras* (Porta et al. 1999). An association of workplace exposure to DDT and the risk of pancreatic cancer is supported by two studies. In a cohort of 5,886 DDT manufacturing plant workers, Garabrant et al. (1992) reported a 7.4-fold higher risk of pancreatic cancer deaths in workers exposed to DDT for an average of 47 months, as determined using work records and interviews with coworkers, compared with workers who had no exposure. Similarly, deaths from pancreatic cancer were significantly higher in a cohort occupationally exposed to DDT compared with a control cohort in an Australian study with follow-up data from 1935 to the 1990s (SMR = 3.57; 95% CI, 1.09–15.40) (Beard et al. 2003). In contrast, other studies have found no association between estimates of DDT exposure among workers (Cocco et al. 2005) or serum/ adipose tissue levels of *p*,*p*′-DDE and pancreatic cancer risk after adjustment for confounders (Hardell et al. 2007; Hoppin et al. 2000). It has been suggested that the etiology of pancreatic cancer may be causally linked to diabetes mellitus and hyperinsulinemia (Michaud 2004) and thus linked to associations between DDT and diabetes (see below).

### Breast cancer

Although neither DDT nor DDE induced mammary tumors in laboratory animal cancer bioassays (IARC 1991), early studies suggested that DDE levels in women were associated with a higher risk of breast cancer (Snedeker 2001). Two recent case–control studies (Charlier et al. 2004; Rubin et al. 2006) also showed higher DDE blood concentrations in cases than controls. However, most case–control studies recently published and reviewed have not supported an association (Brody et al. 2004; Gatto et al. 2007; Ibarluzea et al. 2004; Iwasaki et al. 2008; Lopez-Cervantes et al. 2004; Siddiqui et al. 2005; Snedeker 2001).

The literature on DDT and breast cancer has two main limitations. First, most studies used biological samples collected well after exposure to technical DDT had occurred and relied on serum concentrations of *p*,*p*′-DDE as a proxy for exposure to “DDT.” Second, most studies included women who would not have been exposed to technical DDT when young, yet both animal (Birnbaum and Fenton 2003) and human studies of radiation exposure (Howe and McLaughlin 1996; Tokunaga et al. 1994) strongly suggest that the breast is most vulnerable to environmentally induced carcinogenesis during several critical periods such as *in utero*, before menarche, and before first pregnancy.

Overcoming these past limitations, Cohn et al. (2007) measured concentrations in archived serum samples collected between 1959 and 1967 (peak years of DDT use) from pregnant women participating in the Child Health and Development Studies (CHDS). Medical records were obtained nearly 40 years later. Among women who were = 14 years of age by 1945 (when DDT was first introduced for use by the general public), those with blood concentrations in the highest tertile were five times more likely to develop breast cancer than those with blood levels in the lowest tertile (OR = 5.4; 95% CI, 1.7–17.1). However, there was no association between serum *p*,*p*′-DDT levels and adult risk of breast cancer among women who were not exposed before 14 years of age, and interaction by age in 1945 was statistically significant. This study suggests that the prepubertal and pubertal years are critical periods of exposure. Thus, previous studies that measured exposure in older women may have missed the critical period.

### Other cancers

Research has not supported an association of DDT or DDE and incidence of colorectal, lung, bladder, prostate, endometrial, and stomach cancers (Baris et al. 1998; Cocco et al. 2005; Hardell et al. 2004; Howsam et al. 2004; Purdue et al. 2007; Sturgeon et al. 1998; Weiderpass et al. 2000). Although no associations were found with serum DDT (Rothman et al. 1997), higher DDE levels in dust, adipose tissue, and plasma have been associated with non-Hodgkin lymphoma in case–control studies (Colt et al. 2005; Quintana et al. 2004; Spinelli et al. 2007). For other cancers, such as leukemia (Flodin et al. 1988; Purdue et al. 2007), and testicular cancer (Hardell et al. 2006; McGlynn et al. 2008), evidence remains equivocal.

## Evidence for Diabetes

Since Morgan et al.’s (1980) initial observation that the sum of DDT and DDE levels in serum was 29% higher in occupationally exposed workers with diabetes compared with nondiabetics, a number of other studies have been published on this subject. For example, data from the 1999–2002 NHANES suggested that elevated serum concentrations of *p*,*p*′-DDT (Everett et al. 2007) and *p*,*p*- ′ DDE (Lee et al. 2006) were significantly associated with the prevalence of diabetes. In addition, Lee et al. (2006) reported an increasing trend in the odds of diabetes as exposure to *p*,*p*′-DDE increased (*p*_trend_ < 0.001) with an OR of 4.3 (95% CI, 1.8–10.2) for those = 90th percentile of exposure compared with those in the lowest quartile.

Because diabetes occurs in Mexican Americans twice as frequently as in non- Hispanic whites (Haffner 1998), and serum levels of *p*,*p*′-DDE and *p*,*p*′-DDT are higher in Mexican Americans than other ethnic groups in the U.S. population (CDC 2001), data from the Hispanic Health and Nutrition Examination Survey (HHANES) for 1982–1984 were analyzed. Serum *p*,*p*′-DDT or *p*,*p*′-DDE levels were dose related to the prevalence of self-reported diabetes in Mexican Americans (Cox et al. 2007). Additionally, in a study of Native Americans (Mohawks), Codru et al. (2007) observed a significant positive association between diabetes prevalence and serum levels of *p*,*p*′-DDE.

A Swedish study found that diabetes prevalence was significantly higher (*p*_trend_ = 0.04) in Baltic Sea fishermen with elevated serum DDE levels (Rylander et al. 2005), but not in their wives. With a larger study population (non-fisherman families), the same authors reported a significant positive association (*p*_trend_ < 0.01) between type 2 diabetes and serum *p*,*p*′-DDE levels in Swedish women (Rignell-Hydbom et al. 2007).

Collectively, these studies from the United States and Sweden suggest that body burdens of DDT and/or DDE may be associated with the prevalence of diabetes. A variety of other persistent environmental chemicals also have been associated with diabetes prevalence (Lee et al. 2006). However, given the high correlation among various organochlorine exposures (Bradman et al. 2007), additional research is needed to delineate the specific contributions of DDT and DDE.

## Evidence for Health Consequences to the Fetus

### Pregnancy loss

In the U.S. Collaborative Perinatal Project (CPP), where the median maternal serum DDE level was 24.5 μg/L, high DDE concentrations (45–59 vs. < 15 μg/L) were associated with an increased risk of fetal loss in previous pregnancies (Longnecker et al. 2005). Although the outcome occurred before DDE measurement, this study of 1,717 women corroborated findings from smaller studies (Korrick et al. 2001; Saxena et al. 1981). Venners et al. (2005) studied 338 nulliparous Chinese textile workers with similar DDE concentrations (median, 29 ng/g serum) for the risk of early pregnancy loss (measured by daily human chorionic gonadotropin). Authors reported an OR of 1.17 (95% CI, 1.05–1.29) for each 10-ng/g serum increase in total DDT. In a case– control study of habitual aborters, researchers did not observe higher mean serum DDE levels in cases relative to controls (Sugiura-Ogasawara et al. 2003).

### Gestational length and birth weight

Early studies on DDE and preterm delivery (< 37 weeks of gestation) were small, and results were inconsistent (Berkowitz et al. 1996; O’Leary et al. 1970; Saxena et al. 1981; Wassermann et al. 1982). Studies using data from larger cohorts also have not consistently supported an association between exposure to DDT/DDE and birth weight or gestational duration. In a study of 2,380 pregnant women participating in the CPP, Longnecker et al. (2001) found that the odds of preterm delivery were 3.1 times higher (95% CI, 1.8–5.4) in women with serum DDE = 60 μg/L compared with those with DDE < 15 μg/L during pregnancy. Adjusted odds of having a child small for gestational age also increased, but less consistently (*p*_trend_ = 0.04). However, two analyses of the CHDS, which was conducted around the same time as the CPP, have found no associations of DDT/DDE and preterm delivery or small for gestational age, despite slightly higher median DDE levels than in the CPP (43 vs. 25 μg/L) (Farhang et al. 2005; Jusko et al. 2006).

Most studies of more recent cohorts, which had somewhat lower exposure than those in the earlier studies, did not find an association between maternal serum measurements of DDE and/or DDT and gestational duration, premature labor, birth weight, or other measures of fetal growth such as crown–heel length or head circumference (Bjerregaard and Hansen 2000; Fenster et al. 2006; Gladen et al. 2003; Karmaus and Zhu 2004; Khanjani and Sim 2006; Sagiv et al. 2007; Wood et al. 2007), although some studies did find associations (Siddiqui et al. 2003; Weisskopf et al. 2005; Wolff et al. 2007). The high DDE serum concentrations observed in the CPP and CHDS cohorts during the 1960s are several-fold higher than current serum levels, but substantially lower than in populations where indoor residual spraying is occurring.

### Duration of lactation

Two studies have found a shorter duration of lactation among mothers with high breast milk DDE concentrations (Gladen and Rogan 1995; Rogan et al. 1987). A North Carolina study of 858 women indicated that higher breast milk DDE concentrations were associated with a shorter median duration of lactation (2.5 months for DDE > 6 μg/g lipids vs. 6.5 months for DDE < 1 μg/g lipids) (Rogan et al. 1987). A study of 229 Mexican women found similar results (Gladen and Rogan 1995), but only among women who had previously lactated. Estrogenic effects of DDT were postulated to affect prolactin levels and milk production (Gladen and Rogan 1995; Rogan et al. 1987). A more recent study suggested that DDE serum concentrations were related to decreased rates of breast-feeding initiation as well as shortened duration of lactation in women who had never breast-fed and in non-smoking women (Karmaus et al. 2005b), but a Mexican study found that serum DDE was associated with duration of lactation only in women who previously breast-fed (Cupul-Uicab et al. 2008). In this Mexican study of 784 mothers of male term babies, the hazard ratio of weaning for women with high serum DDE levels (cutoff point, 9 μg/g lipids) was 1.76 (95% CI, 1.22–2.53) in women who had previously breast-fed and 0.91 (95% CI, 0.66–1.26) in women who had never breast-fed. Previous lactation can reduce the maternal body burden of DDE, and women who breast-feed longer for previous infants tend to do so for the current baby. Thus, associations between DDE levels and early weaning may be spurious, and further research is warranted.

### Urogenital birth defects

Studies in rats have suggested a relationship between fetal tissue concentrations of 10–20 ppm of *p*,*p*′-DDE and reproductive abnormalities in male offspring (Gray et al. 2001). In the CPP birth cohort, Longnecker et al. (2002) reported ORs of 1.07 (95% CI, 0.97–1.18) for cryptorchidism (*n* = 219), 1.01 (95% CI, 0.90–1.14) for hypospadias (*n* = 199), and 1.06 (95% CI, 0.97–1.16) for polythelia (*n* = 167) for each 2.67 μg/g lipid increase in maternal serum *p*,*p*′-DDE (Longnecker et al. 2002). However, in the CHDS birth cohort, Bhatia et al. (2005) found that the odds of cryptorchidism were twice as high in participants with *p*,*p*′-DDT levels above versus below the median (OR = 1.97; 95% CI, 1.40–2.54) but found no associations between maternal serum *p*,*p*′-DDE and the odds of cryptorchidism, or between *p*,*p*′-DDT and *p*,*p*′-DDE concentrations and odds of hypospadias. A Spanish study also reported more than a doubling of the odds of cryptorchidism and/or hypospadias cases associated with detectable levels of *p*,*p*- ′ DDT (OR = 2.63; 95% CI, 1.21–5.72) and *o*,*p*′-DDT (OR = 2.25; 95% CI, 1.03–4.89) measured in placental tissues (Fernandez et al. 2007). Other studies conducted in France (Brucker-Davis et al. 2008) and in Finland and Denmark (Damgaard et al. 2006) reported increased colostrum levels of *p*,*p*′-DDE, and elevated breast milk concentrations of *p*,*p*′-DDT, *o*,*p*′-DDT, *p*,*p*′-DDE, and *p*,*p*′-dichloro-diphenyldichloroethane (DDD), respectively, in cases of cryptorchidism compared with controls, although associations were not statistically significant.

### Child growth

Evidence for an association between physical growth after birth and DDT and DDE exposure is inconsistent. Maternal DDT concentrations were not associated with child weight or height at 5 years of age in children participating in the CHDS (Jusko et al. 2006). In contrast, those who had the highest prenatal concentrations of DDE in the CPP cohort (= 60 μg/L) compared with the lowest (< 15 μg/L) were significantly shorter at 1, 4, and 7 years of age (Ribas-Fito et al. 2006a). However, a study that examined growth in adolescent boys participating in the CPP found no relation between maternal serum levels of DDE or DDT in pregnancy and height, body mass index (BMI), and other measures of growth (Gladen et al. 2004). Two other studies in older children and in populations with much lower serum levels reported conflicting findings: A study from Germany found shorter height in 8-year-old girls in relation to higher DDE concentration (Karmaus et al. 2002), and an older North Carolina study found a relation to taller height in boys 12–14 years of age (Gladen et al. 2000). Overall, the evidence for the relation of maternal DDT exposure and child physical growth is weak.

## Evidence for Reproductive Effects

### Age of onset of puberty

A small number of human studies have examined DDT/DDE and onset of menarche in girls, with two studies finding associations of earlier age at menarche with higher exposure (Ouyang et al. 2005; Vasiliu et al. 2004) and one study finding no association (Denham et al. 2005). Ouyang et al. (2005) found that women with higher serum levels of total DDT (i.e., the sum of *p*,*p*′- and *o*,*p*′-isomers of DDT, DDE, and DDD) measured in adulthood (mean age, 24.9 years) reported significantly earlier age at onset of menarche, adjusting for BMI and birth year. In contrast, Denham et al. (2005) found no association between concurrent DDE blood concentrations [geometric mean (GM) = 0.35 ppb] and menarcheal status among 138 Mohawk girls 10–16.9 years of age. Vasiliu et al. (2004) estimated serum DDE levels during pregnancy in 151 women by back-calculating from measurements made up to 25 years later. They reported that higher (estimated) prenatal DDE levels were associated with earlier age at menarche in daughters (adjusted β = −0.07, *p* = 0.04).

Two studies examined onset of puberty rather than menarche in relation to DDE exposure. Gladen et al. (2000) found no association between transplacental or lactational DDE concentrations and pubertal stage as self-reported in a cohort of approximately 315 adolescent girls with relatively high exposure (range, 0.3–25.8 ppm). Wolff et al. (2008) performed Tanner exams on approximately 80 9-year-old girls and found no difference in concurrent DDE plasma levels in those girls who had reached Tanner breast stage 2 (onset of puberty) and those who did not, but DDE levels were low. Only one study has examined onset of puberty in boys. Gladen et al. (2000) found no association between DDE and self-reported Tanner staging in boys up to 16 years of age. All these studies examined associations with DDE and not DDT.

Although these studies of puberty and menarche suggest an association with exposure, no study has examined the relationship of serum levels of DDE and DDT concentrations in blood collected before puberty in relation to Tanner staging in girls or boys.

### Male fertility

Researchers have investigated the seminal parameters of men living in regions of high DDT use. In one such study (de Jager et al. 2006), participants were drawn from rural communities in the malaria-endemic region of Chiapas, Mexico, with a history of high use and where DDT was sprayed inside their homes at least annually from the late 1940s until 1997 (Stapleton 1998); sampling took place between 2000 and 2001. The mean serum DDE concentration (45 ± 31 μg/g lipids) was 100 times higher than reported in unexposed populations. The percentage of motile sperm was negatively correlated with plasma DDE concentrations, whereas the percentage of sperm with morphologic tail defects and insufficient sperm chromatin condensation was positively correlated with these levels (de Jager et al. 2006).

In a study conducted in Limpopo, South Africa, men were selected from rural communities in a malaria-endemic area where DDT is sprayed annually inside unpainted houses, but not inside painted houses (Aneck-Hahn et al. 2007). The GM serum concentrations of DDT (90.2 ± 102.4 μg/g) and DDE (215.5 ± 210.6 μg/g) in the 311 participants from this area were extremely high. DDT and DDE serum concentrations were also significantly higher (*p* < 0.001) in participants whose houses were sprayed with DDT (101.9 μg/g lipid DDT and 239.0 μg/g lipid DDE) compared with those whose houses were not sprayed (30.5 μg/g lipid DDT and 99.5 μg/g lipid DDE). Their semen volume was low (1.9 ± 1.3 mL) and several sperm motion parameters were impaired, including the percentage of motile sperm in men with higher DDT (*r* = −0.27, *p* < 0.001) and DDE concentrations (*r* = −0.20, *p* < 0.001). In another cross-sectional study of 48 DDT applicators (Dalvie et al. 2004) conducted in Limpopo, overall semen quality was low, and DDT, but not DDE, serum levels were associated with decreased sperm count.

Studies of semen quality or genetic markers in sperm have been conducted in other populations, usually with lower exposure than noted in Chiapas or South Africa. For example, case–control studies of men of sub-fertile couples have found no difference in the men’s DDE serum levels (Charlier and Foidart 2005) but higher current blood levels in the mothers of the cases compared with controls, and no association with DNA integrity (neutral comet assay) (Hauser et al. 2003). Similarly, the multinational (Greenland; Warsaw, Poland; Kharkiv, Ukraine; and Sweden) INUENDO study of European and Inuit pregnant women and their spouses failed to find associations of DDE serum levels and conventional measures of semen quality (Toft et al. 2006), chromatin integrity (Spano et al. 2005), and hormone levels or a measure of apoptosis (Stronati et al. 2006). However, as in many populations where exposure to several organochlorines, including polychlorinated biphenyls, may be highly correlated, independent associations with DDE often could not be established.

Overall, studies of highly exposed populations suggest that male fertility may be adversely affected by DDT exposure, but studies in populations with moderate to low exposure levels do not support a relationship between exposure and male fertility outcomes.

### Female reproduction, fertility, and time to conception

Two recent studies examined the relationship of DDT/DDE levels and menstrual cycle characteristics and found only weak associations (Chen et al. 2005; Cooper et al. 2005). However, one study from the INUENDO population reported a 3-fold increase in risk of long menstrual cycles among Polish women but not among women from other European countries (Toft et al. 2008). Another study using data from the Hispanic Health and Nutrition Examination Survey found that both DDT and DDE levels were associated with a significantly earlier age of menopause (Akkina et al. 2004).

Two cross-sectional studies found suggestive evidence that *p*,*p*′-DDE serum levels in pregnant women were correlated with delays in conception (Axmon et al. 2006; Law et al. 2005), but another study in Mexican-American women did not find a relation with either *p*,*p*′-DDT or *p*,*p*′-DDE serum levels (Harley et al. 2008). One of these studies (Axmon et al. 2006) also examined paternal serum *p*,*p*′-DDE and found no effects on partners’ time to pregnancy. In contrast, a study of 105 male DDT applicators found that *p*,*p*′-DDE exposure, estimated by occupation history, was associated with delayed time to pregnancy in their spouses, defined using marriage dates and birth dates of first-born children (Cocco et al. 2005).

The only study to examine *in utero* exposure to DDT and time to pregnancy was conducted among 289 women born in California between 1960 and 1963 (CHDS). Authors found that each 10-μg/L increase in mothers’ serum concentrations of *p*,*p*′-DDT and *p*,*p*′-DDE during pregnancy was associated with a 32% reduction and a 16% increase, respectively, in their daughters’ per cycle probability of pregnancy (Cohn et al. 2003). The ratio of these two compounds varied considerably, and longer time to pregnancy in daughters was observed as the ratio of *p*,*p*′-DDT to *p*,*p*′-DDE increased in maternal serum samples. Findings suggest that recent exposure or direct exposure to the pesticide, rather than chronic exposure to *p*,*p*′-DDE in the food chain, was the underlying risk factor.

Overall, the few studies conducted to date suggest that DDT exposure may affect time to pregnancy, but more research is needed.

## Evidence for Neurodevelopmental Effects

DDT exerts its insecticidal effects by disrupting the nervous system. Animal studies confirm that DDT is a neurodevelopmental toxicant (ATSDR 2002). In mice, exposure to DDT timed to sensitive periods of pre-natal (Craig and Ogilvie 1974) and neonatal (Eriksson and Nordberg 1986; Eriksson et al. 1990; Johansson et al. 1996) nervous system development has been shown to cause behavioral and neurochemical changes into adulthood.

The few studies conducted in humans have focused primarily on exposure to DDE rather than DDT. In a North Carolina birth cohort recruited in the 1980s, Rogan et al. (1986) reported that maternal serum and breast milk DDE levels were related to hyporeflexia in a dose-dependent fashion in infants assessed by the Brazelton Neonatal Behavioral Assessment Scale (BNBAS), but this finding has not been replicated in more recent studies (Engel et al. 2007; Fenster et al. 2006; Stewart et al. 2000). In a recent investigation from Massachusetts, DDE measured in cord blood was negatively related to BNBAS measures of alertness and attention and positively related to measures of irritability, although only the trend for irritability was significant (Sagiv et al. 2008).

The North Carolina study reported no adverse association between perinatal DDE exposure and performance on the Bayley Scales of Infant Development (BSID) from 6 to 24 months of age (Gladen et al. 1988; Rogan and Gladen 1991); on the McCarthy Scales of Children’s Abilities (MCSA) at ages 3, 4, and 5 years; or on school performance at 8–10.5 years (Gladen and Rogan 1991). Similarly, a study conducted in Oswego, New York, found no association of DDE levels and performance on the Fagan Test of Infant Intelligence at 6 and 12 months of age (Darvill et al. 2000). However, a smaller Spanish study (Ribas-Fito et al. 2003) of 92 infants 13 months of age found a significant negative association between relatively low cord serum DDE levels and cognitive, psychomotor, and social development on the BSID and Griffith Scales of Infant Development. Similarly, a study of 230 infants from Mexico found that maternal serum DDE levels were inversely associated with psychomotor development scores on the BSID at 3, 6, and 12 months (Torres-Sanchez et al. 2007). Using the Center for the Health Assessment of Mothers and Children of Salinas (CHAMACOS) cohort of Mexican-American children, Eskenazi et al. (2006) also reported an inverse association of maternal serum DDE and psychomotor development at 6 and 12 but not at 24 months, and with mental development at 24 months.

In a small study measuring visual evoked potentials (VEPs) in 12-month-olds (Riva et al. 2004), wave latency VEPs at 15 min were significantly related to the colostral levels of both DDT and DDE, and wave latency VEPs at 60 min were related to levels of DDT. However, findings were no longer statistically significant after authors controlled for the plasma levels of long-chain polyunsaturated fatty acids, thought to be one of the beneficial components of breast milk for brain development. The authors concluded that breast-feeding in itself may exert a protective effect against contaminants in human milk.

Only two studies have examined the relationship of DDT levels and cognitive functioning. Increased maternal serum DDT levels were associated with poorer psychomotor development at 6, 12, and 24 months and mental development at 12 and 24 months in the CHAMACOS cohort (Eskenazi et al. 2006). Breast-feeding did not have a negative relationship with mental development in the group with the highest maternal DDT levels. In another study, cord serum DDT levels were also found to be associated with poorer performance in general cognitive, memory, quantitative, verbal, and executive function domains of the MCSA in 4- and 5-year-old children in Spain (Ribas-Fito et al. 2006b).

These studies suggest that DDT, and less so DDE, may be associated with neurodevelopmental deficits. In addition, breast-feeding may modulate some of the negative effects of DDT, but this needs to be examined when exposure is high such as in communities where indoor residual spraying is occurring. Follow-up studies of these populations are needed to verify whether these developmental deficits persist.

## Evidence for Other Health Effects

Although thyroid hormones are essential for normal brain development (Dunn 1993), studies suggest that DDE, and possibly DDT, may depress triiodothyronine (T_3_) and/or thyroxine (T_4_) in maternal, cord, and preschool children’s blood (Abdelouahab et al. 2008; Alvarez-Pedrerol et al. 2008; Asawasinsopon et al. 2006; Maervoet et al. 2007; Takser et al. 2005) but not entirely consistently (Chevrier et al. 2008). Higher DDT levels were also found in cases of congenital hypothyroidism relative to controls (Nagayama et al. 2007a). Results from studies conducted in men and nonpregnant women have generally been inconsistent. Two of these studies reported a positive association between serum DDE and thyroid-stimulating hormone (TSH) concentrations (Meeker et al. 2007; Rylander et al. 2006), one found a positive correlation between DDE and total T_3_ (Langer et al. 2007a), whereas other studies found no associations between DDE and TSH (Langer et al. 2005, 2006, 2007a, 2007b; Turyk et al. 2006), total T_3_ or the free T_4_ index (Turyk et al. 2006).

DDT and particularly DDE have demonstrated the potential for modulating the human immune response as measured by multiple markers such as interleukin-4 (Daniel et al. 2002) and interleukin-13 (Brooks et al. 2007), plasma levels of type 1 (interferon-γ) response to mitogen in nursing mothers, white blood cell counts, and various lymphocyte phenotypes (Nagayama et al. 2007b; Noakes et al. 2006; Vine et al. 2001), and immunoglobulin (Ig) A, G, and E levels (Cooper et al. 2004; Karmaus et al. 2005a). Associations with immune system–related conditions such as aplastic anemia (with DDT but not DDE) (Ahamed et al. 2006; Issaragrisil et al. 2006), asthma (with DDE) (Karmaus et al. 2001; Sunyer et al. 2005), otitis media (with DDE) (Dallaire et al. 2004), and farmer’s lung (with technical DDT use) (Hoppin et al. 2007) were also reported. In children, one cross- sectional study of German schoolchildren found that DDE levels in blood were associated with increased IgE blood levels and asthma (Karmaus et al. 2001). A longitudinal study of 405 Spanish children confirmed the association between DDE exposure and asthma, but found that DDE was not associated with IgE levels (Sunyer et al. 2005). Additional research is needed to understand the effects of DDT/ DDE on the immune system and associated diseases, especially because DDT is used in areas where there are often high rates of HIV.

## Conclusions

The use of DDT historically may have helped prevent millions of infections and deaths from insect-borne diseases. Based on recent studies, we conclude that humans are exposed to DDT and DDE, that indoor residual spraying can result in substantial exposure, and that DDT may pose a risk for human populations. However, few studies have measured body burdens of both DDE and DDT, and studies have rarely investigated the effects of DDT/DDE exposure at levels observed in populations exposed through indoor residual spraying. Furthermore, information on exposure to DDT/DDE during critical periods is limited for outcomes such as cancer.

We are concerned about the health of children and adults given the persistence of DDT and its active metabolites in the environment and in the body, and we are particularly concerned about the potential effects of continued DDT use on future generations. We recognize the serious implications of restricting DDT use given that an estimated 880,000 people die each year from malaria, most of whom are < 5 years of age (WHO 2008). Given our continually deepening understanding of the effects of DDT use on humans, we ask global policy makers to consider the following issues:

- In the United States, individuals have been exposed to DDT by working in occupational settings and by living in proximity to DDT manufacturing facilities. State and federal agencies should monitor levels of contaminants in residents near Superfund sites (e.g., Pine River/Velsicol Chemical Corp. Michigan Superfund site) and conduct health effects studies if biomonitoring indicates persistently elevated levels of DDT or DDE.
- Few studies of health outcomes have been conducted in populations where indoor residual spraying with DDT is occurring. These populations likely have much higher exposures to DDT and may differ from those previously studied in ways that might affect susceptibility (e.g., genetics, diet, health status, and social class). Research is needed to determine the exposure and health risks associated with DDT used for indoor residual spraying in the relevant communities.
- Children, pregnant women, and those who are immunocompromised may be most at risk for the effects of DDT. People in many malaria-endemic areas where DDT is being used also have high rates of HIV/AIDS infection.
- Breast-feeding is the best form of nutrition for infants and is recommended up to at least 1 year of age by the American Academy of Pediatrics (2005). In some African countries, women may breast-feed for up to 2 years. However, because of the lipophilic nature of DDT/DDE, breast milk is a major route of DDT/DDE exposure to infants. Significant public health consequences could ensue should breast-feeding be discouraged as a result of high DDT contamination.
- DDT may be a valuable short-term approach for controlling malaria, but measures should be taken to reduce human exposure to this pesticide. DDT exposure could conceivably be reduced through strict adherence to indoor residual spraying guidelines, better education of communities and applicators regarding the potential hazards of DDT exposure, improved application methods and formulations, and a better understanding of the determinants of exposure.
- New methods of vector control should be developed and rigorously tested considering local differences such as in vectors, parasites, ecology, and culture. As is the case for DDT, new methods for vector control should be evaluated not only for their effectiveness, but also for their potential adverse effects on the environment and populations, including to susceptible subpopulations such as those who are immunocompromised and malnourished. For example, pyrethroids have been substituted for DDT in indoor residual spraying in some locations, yet there is little information on their effects to human health.

Current evidence on DDT exposure to human populations and on its potential health effects support the Stockholm Convention on Persistent Organic Pollutants, which emphasizes that DDT should be used with caution, only when needed, and when no other effective, safe, and affordable alternatives are locally available. Under the convention, each country currently using DDT is required to provide an implementation and management plan to limit the use of DDT to disease vector control and to reduce reliance on DDT. Countries should be assisted so that they can ultimately rely on other sustainable methods, techniques, and strategies for malaria control. Given the paucity of data in populations who are currently potentially exposed to high levels of DDT, we urge the global community to monitor exposure to DDT and to evaluate its potential health impacts both in malaria-endemic regions of the world and in locations where DDT use has been historically high, such as the Pine River Superfund site.

## Figures and Tables

**Table 1:** Median *p*,*p*′-DDE and *p*,*p*′-DDT serum concentrations in pregnant women or women of reproductive age from various study populations.

Location	Years	*p,p*′-DDE (μg/g lipids)	*p,p*′-DDT (ng/g lipids)	Reference
Canada, Nunavik	1994–1997	0.2^a^	15^a^	Van Oostdam et al. 2004
Canada, Kitikmeot	1994–1997	0.1^a^	8.3^a^	Van Oostdam et al. 2004
China, Anhui Province	1996–1998	5.9^b^	—	Perry et al. 2005
Finland	1994–1997	0.06^a^	2.4^a^	Van Oostdam et al. 2004
Greenland	1994–1997	0.4^a^	26^a^	Van Oostdam et al. 2004
Greenland	2002–2003	0.3	—	Jonsson et al. 2005
Iceland	1994–1997	0.1^a^	4^a^	Van Oostdam et al. 2004
Mexico, Chiapas	1998	4.8	676	Koepke et al. 2004
Mexico, Chiapas	2002–2003	2.7	250	Longnecker et al. 2007
Mexico, Morelos	2001–2005	1.0^a^,^c^	—	Torres-Sanchez et al. 2007
Mexico, Oaxaca	2000	8.0^a^	3,140^a^	Barraza-Vazquez et al. 2008
Norway	1994–1997	0.1^a^	3^a^	Van Oostdam et al. 2004
Poland, Warsaw	2003–2004	0.4	—	Jonsson et al. 2005
Poland, Wielkopolska	2004	0.3	20.2	Jaraczewska et al. 2006
Russia	1994–1997	0.4^a^	48^a^	Van Oostdam et al. 2004
Spain	NR	4^b^,^c^	1,330^b^,^c^	Jimenez Torres et al. 2006
Sweden	1994–1997	0.1^a^	2.4^a^	Van Oostdam et al. 2004
Sweden, Uppsala County	1996–1999	0.01^b^	5^b^	Glynn et al. 2007
Sweden	2002–2003	0.1	—	Jonsson et al. 2005
Ukraine, Kharkiv	2002–2004	0.7	—	Jonsson et al. 2005
USA, Philadelphia, PA	1959–1965	5.7	1,900	Gladen et al. 2004
USA, Multicenter	1959–1965	3.2^c^	—	Longnecker et al. 2005
USA, California	1959–1967	2.2	2,000	Bhatia et al. 2005
USA, Alaska	1994–1997	0.1^a^	3.7^a^	Van Oostdam et al. 2004
USA, California	1999–2000	1.1	12.5	Bradman et al. 2007
U.S. representative sample	2001–2002	0.1	< 17.4	CDC 2005

Abbreviations: —, not measured; NR, not reported.

a Geometric mean.

b Arithmetic mean.

c Calculated assuming third-trimester serum lipid levels of 7.9 g/L (Longnecker et al. 2003).
